# Interleukin-27 Is Differentially Associated with HIV Viral Load and CD4^+^ T Cell Counts in Therapy-Naïve HIV-Mono-Infected and HIV/HCV-Co-Infected Chinese

**DOI:** 10.1371/journal.pone.0096792

**Published:** 2014-05-09

**Authors:** Lai He, Jin Zhao, Maggie Haitian Wang, Kenny K. Y. Siu, Yong-Xia Gan, Lin Chen, Benny C. Y. Zee, Li Yang, Hsiang-Fu Kung, Zheng-Rong Yang, Ming-Liang He

**Affiliations:** 1 Stanley Ho Center for Emerging Infectious Diseases, School of Public Health and Primary Care, the Chinese University of Hong Kong, Hong Kong SAR, China; 2 Shenzhen Center for Disease Control and Prevention, Shenzhen, China; 3 Division of Biostatistics, School of Public Health and Primary Care, the Chinese University of Hong Kong, Hong Kong SAR, China; 4 Division of Biosciences, Faculty of Life Science, University College London, London, United Kingdom; 5 Division of Digestive Diseases, West China Hospital, Sichuan University, Chengdu, China; 6 Li Ka Shing Institute of Health Sciences, School of Public Health and Primary Care, the Chinese University of Hong Kong, Hong Kong SAR, China; Institute of Infection and Global Health, United Kingdom

## Abstract

Human Immunodeficiency Virus (HIV) infection and the resultant Acquired Immunodeficiency Syndrome (AIDS) epidemic are major global health challenges; hepatitis C virus (HCV) co-infection has made the HIV/AIDS epidemic even worse. Interleukin-27 (IL-27), a cytokine which inhibits HIV and HCV replication *in vitro*, associates with HIV infection and HIV/HCV co-infection in clinical settings. However, the impact of HIV and HCV viral loads on plasma IL-27 expression levels has not been well characterized. In this study, 155 antiretroviral therapy-naïve Chinese were recruited. Among them 80 were HIV- and HCV-negative healthy controls, 45 were HIV-mono-infected and 30 were HIV/HCV-co-infected. Plasma level HIV, HCV, IL-27 and CD4+ number were counted and their correlation, regression relationships were explored. We show that: plasma IL-27 level was significantly upregulated in HIV-mono-infected and HIV/HCV-co-infected Chinese; HIV viral load was negatively correlated with IL-27 titer in HIV-mono-infected subjects whereas the relationship was opposite in HIV/HCV-co-infected subjects; and the relationships between HIV viral loads, IL-27 titers and CD4^+^ T cell counts in the HIV mono-infection and HIV/HCV co-infection groups were dramatically different. Overall, our results suggest that IL-27 differs in treatment-naïve groups with HIV mono-infections and HIV/HCV co-infections, thereby providing critical information to be considered when caring and treating those with HIV mono-infection and HIV/HCV co-infection.

## Introduction

HIV causes progressive failure of the immune system which eventually leads to AIDS, and it is characterized by susceptibility to opportunistic infections and tumors. Since the first report of AIDS in 1981 and the discovery of causative agent HIV in 1983 [1]–[3], HIV/AIDS has caused nearly 30 million deaths worldwide [4]. Although drug cocktails used in highly active antiretroviral therapy (HAART) significantly slow the progression of HIV-infected individuals to AIDS, HIV/AIDS is still one of the leading causes of death. In 2009, an estimated 33.3 million people were living with HIV/AIDS and about 1.8 million people died of AIDS-related diseases worldwide [4]. Along with the rapid economic development in China, the severity of the country's HIV/AIDS epidemic is also growing. The rapidly increasing HIV prevalence in China has shifted from injection drug users (IDUs) and former blood donors into men who have sex with men and female sex workers [5]–[7]. HCV infection makes the HIV/AIDS epidemic much worse; this is because HCV co-infection facilitates HIV disease progression and increases the morbidity and mortality of AIDS patients [8]–[10]. Upon HIV infection, the human immune system responds to restrict, inhibit and destroy HIV through different mechanisms. Among the system, IL-27 is an attractive cytokine belonging to the IL-12 family with important implications in Th1 responses [11]. Composed of EBI3 and p28 protein, IL-27 plays pivotal roles both in pro-inflammatory and anti-inflammatory responses [11]–[14]. *In vitro* experiments demonstrate that IL-27 inhibits ×4 and R5 HIV replication in peripheral blood mononuclear cells (PBMC), CD4^+^ T cells and monocyte-derived macrophages (MDMs) through the induction of type I interferon and activation of multiple interferon-inducible genes [15]–[18]. In a cultured cell system, IL-27 induces IFN-γ-like signals and induction of antiviral responses in hepatoma cells, hepatocytes and hepatic stellate cells [19], [20]. It also inhibits HCV in a dose-dependent manner, indicating that IL-27 may be a potential therapeutic cytokine for HCV and HIV/HCV co-infection [21], [22].

Clinical data on serum IL-27 in HIV/AIDS patients are very limited. It was reported that IL-27 was negatively correlated with HIV viral load, downregulated under HCV co-infection, and related to the CD4^+^ T cell counts [23], [24]. In a previous study, we found that the plasma IL-27 level in HIV-positive Chinese was significantly higher than in the healthy controls, and that IL-27 titer was positively correlated with CD4^+^ T cell counts among HIV positive, antiretroviral therapy-naïve Chinese [25]. In this study, we aim to explore how the co-infection of the HIV and HCV virus affects serum IL-27 titer among treatment-naïve subjects.

## Material and Methods

### Study participants

Subjects were recruited from an ongoing voluntary-based HIV/AIDS surveillance study in Shenzhen, an immigrant city in Guangdong province, China, from September 2009 to December 2010 [26], [27]. Written consents were obtained from participants, and the interviews were conducted by experienced research staff. All participants were Chinese and they were screened for HIV, HBV and HCV infections. Confirmed HIV-positive individuals were referred to HIV prevention, treatment, and care programs. HBV- and HCV-positive participants were referred to local hospitals for treatment. All infections were recorded following the requirements relating to reportable infectious diseases. The study protocol was approved by the Shenzhen Center for Disease Control and Prevention and the Chinese University of Hong Kong.

### HIV, HBV and HCV serological assays

Whole blood specimens were collected with EDTA anticoagulant and centrifuged at 1,000 rpm for 10 minutes; plasma was then gathered and stored at −80°C until analysis. Plasma was screened by ABBOTT PRISM HIV O Plus (Abbott Laboratories, IL, USA) and ELISA (Beijing Wantai Biological Pharmacy Enterprise Co., Ltd, Beijing, China) for HIV infection, and positive samples were further confirmed by HIV-1/2 Western blot assay (HIV Blot 2.2 WB; Genelabs Diagnostics, Singapore) [26], [27]. HBV and HCV infections were determined by HBsAg ELISA kit (Beijing Wantai Biological Pharmacy Enterprise Co., Ltd, Beijing, China) and Antibody to hepatitis C virus (HCV) ELISA kit (Beijing Wantai Biological Pharmacy Enterprise Co., Ltd, Beijing, China), in accordance with the manufacturers' protocols, respectively. Individuals infected with HBV were excluded from this study.

### CD4^+^ counting

CD4^+^ T cell counts in fresh whole blood were assessed by a three-color fluorescence kit for CD4/CD8/CD3 (BD Biosciences, San Jose, CA, USA) on FACS Calibur flow cytometer (BD Biosciences, San Jose, CA, USA) within eight hours. Multiset software was used for CD4/CD8/CD3 counts analysis [26], [27]. Individuals with CD4^+^ counts of less than 350 cells/µL were considered as being treated for AIDS and they were excluded from this study.

### Plasma IL-27 level

Plasma IL-27 level was measured with pre-coated LEGEND MAX Human IL-27 ELISA Kit (BioLegend Inc., San Diego, CA, USA) in accordance with the manufacturer's instructions. Standard curve was generated by using serial diluted standards. Each plate was run on an independent standard curve for reference. Plasma IL-27 concentration was calculated by generated standard curve. The minimum detectable level of the ELISA kit was 11 pg/ml and the kit had no cross-reaction with 14 other human cytokines [25].

### Quantification of HIV, HCV viral loads

HIV and HCV viral loads were measured among HIV-mono-infected individuals and HIV/HCV-co-infected individuals. The plasma HIV RNA level was measured with quantitative reverse polymerase chain reaction (COBAS AMPLICOR HIV-1 Monitor Test version 1.5, Roche Molecular Systems, Branchburg, NJ, USA) following the manufacturer's instructions. The kit detects HIV-1 RNA ranges over 48 to 10,000,000 copies/ml. Plasma HCV RNA was quantified by commercial quantitative RT-PCR kit (Shenzhen PG Biotech Co., Ltd. Shenzhen, China), with a detection linear range of 10^3^–10^7^ IU/ml. Dilution was conducted whenever necessary.

### Statistical analysis

Data was analyzed with SPSS version 20.0 (SPSS Inc., Chicago, IL, USA). *T* test was used for the comparison of plasma IL-27 between HIV-infected samples and healthy controls. Pearson's correlation co-efficient (r) was applied to test the correlation between plasma IL-27 titer, CD4^+^ T cell counts, and HIV viral loads. Because the HIV titers (copies/ml) and HCV titers (IU/ml) have exponential trends of increase, the natural logarithm is taken on these variables for calculating correlation with other variables. A *P* value of less than 0.15 was considered a trend of clinical relevance [23], and a p-value of less than 0.05 was designated as statistically significant.

## Results

### Participants' demographics

In this study, 155 antiretroviral therapy-naïve Chinese were recruited. Among them 80 were HIV- and HCV-negative healthy controls, 45 were HIV-mono-infected and 30 were HIV/HCV-co-infected individuals. The average ages of the healthy group, the HIV mono-infection group and the HIV/HCV co-infection group were 28 years, 30 years and 32 years, respectively (Table 1). In the HIV mono-infection and the HIV/HCV co-infection groups, the male percentages were 84.4% and 90.0%, respectively, and in the healthy control group, 75.5% were male. All HIV-positive individuals had not yet developed AIDS-defining symptoms and were antiviral therapy-naïve.

**Table 1:** Demographic characteristics of participants.

Characteristics	Control	HIV mono-infection	HIV/HCV co-infection
Number	80	45	30
Age (years)	28	30	32
Gender (male/female)	62/18	38/7	27/3
IL-27 titer (mean ± SD, pg/ml)	410±226	675±334	678±300
CD4 count (mean ± SD, cells/µl)		412.2±229.4	399.9±208.4
HIV viral load (copies/ml)			
Median		25,800	38,100
Range		338–578,000	260–446,000

### IL-27 and CD4+ level in HIV-positive individuals

**Figure 1:**
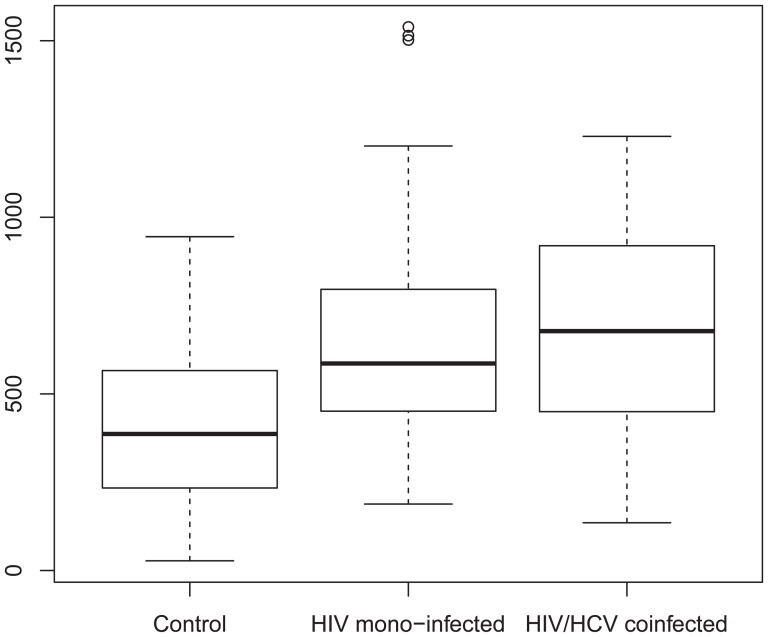
IL-27 titers in HIV-positive individuals and controls. Plasma IL-27 concentration was measured in 80 healthy controls, 45 HIV-mono-infected, and 30 HIV/HCV-co-infected individuals by ELISA. Box-plot illustrated the first quartile, median and third quartile; the ends indicate 5% and 95% percentiles.

The IL-27 levels' ranges were 27 pg/ml to 890 pg/ml 188 pg/ml to 1,539 pg/ml and 135 pg/ml to 1,229 pg/ml in the control group, HIV mono-infected group, HIV/HCV-co-infected group, respectively. IL-27 titer was significantly upregulated in the HIV-infected individuals compared to healthy controls, as shown in the box-plot of Figure 1 (HIV mono-infection versus control: 675±334 pg/ml versus 410±226 pg/ml, mean ± SD, *P*<0.001; HIV/HCV co-infection versus Control: 678±300 pg/ml versus 410±226 pg/ml, *P*<0.001); there was, however, no statistical difference between the HIV mono-infection and the HIV/HCV co-infection individuals (675±334 pg/ml versus 678±200 pg/ml). CD4^+^ counts in whole blood cells without differentiation of HCV- or HIV-specific were measured, and individuals who had CD4^+^ counts less than 350 cells/µL were excluded. No significant difference was observed for the CD4^+^ counts in the HIV mono-infected and the HIV/HCV co-infected groups (412.2±229.4 counts versus 399.9±208.4 counts; Table 1).

### The negative association between CD4^+^ counts and HIV viral loads was weaker in the HCV/HIV-co-infected group

We found that the CD4^+^ counts were significantly negatively associated with the HIV viral loads in the mono-infected HIV group (*p* = 0.018; Table 2). While in the co-infected subjects, their correlation was −0.09 (*p* = 0.323; Table 3), which still preserved a negative relationship, but the magnitude of the correlation was much weaker than in the mono-infected group (*r* = −0.54, *p* = 0.0045 single tailed test). The result was consistent to the primary outcome of HIV infection in that the CD4^+^ cell counts continued to decline, thereby resulting in AIDS. However, the strength of their association was weakened by the co-infection with an HCV virus that at the same time facilitated disease progression and mortality.

**Table 2:** Pairwise correlation of variables in the HIV-mono-infected group (N = 43).

Variable 1	Variable 2	Correlation co-efficient (r)	T-statistic	P-value
CD4	log(HIV)	−0.31	−2.17	0.018
CD4	IL-27	0.17	1.14	0.131
IL-27	log(HIV)	−0.04	−0.28	0.392

**Table 3:** Pairwise correlation of variables in the HIV/HCV-co-infected group (N = 30).

Variable 1	Variable 2	Correlation co-efficient (r)	T-statistic	P-value
CD4	log(HIV)	−0.09	−0.47	0.323
CD4	IL-27	−0.20	−1.07	0.147
IL-27	log(HIV)	0.36	2.05	0.025
IL-27	log(HCV)	−0.13	−0.68	0.251
CD4	log(HCV)	−0.13	−0.70	0.244
log(HIV)	log(HCV)	−0.16	−0.88	0.193

### CD4^+^ counts differentially regulated by IL-27 in the HIV-mono-infected group and the HIV/HCV-co-infected group

**Figure 2:**
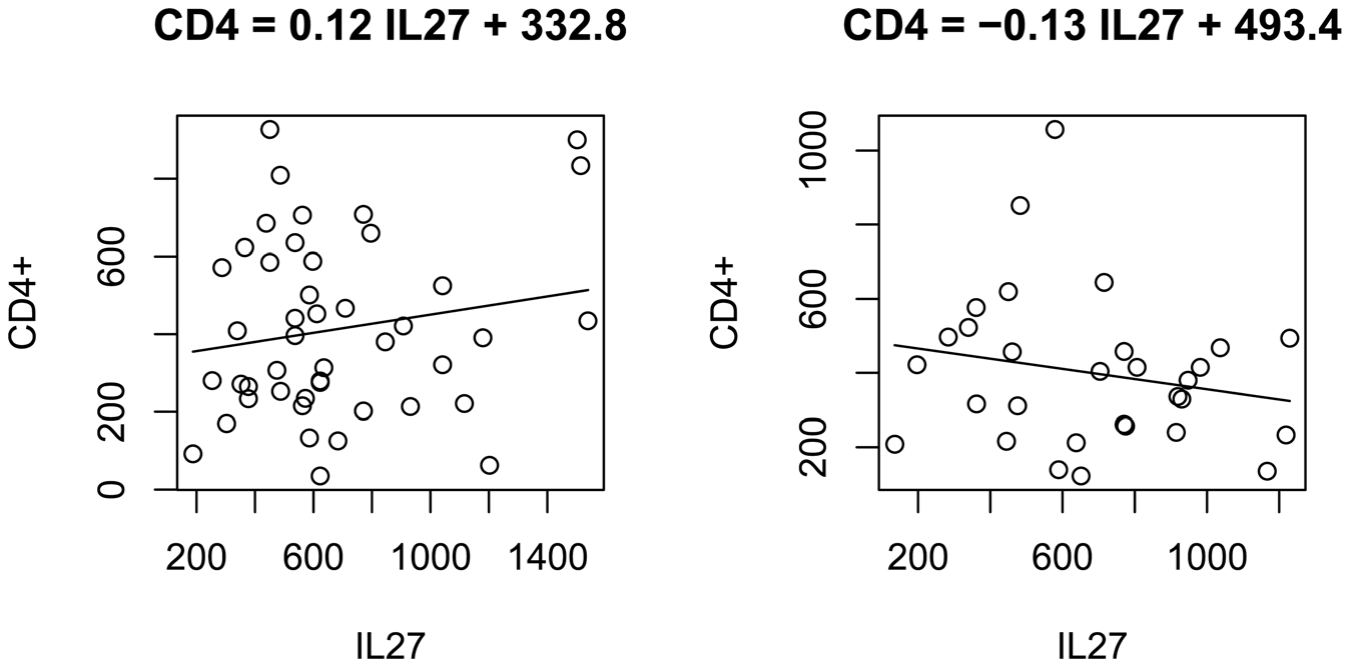
A. Linear regression of CD4^+^ against IL-27 in the HIV-mono-infected group. In HIV-mono-infected individuals, the CD4^+^ counts had a positive relationship with IL-27. The linear regresion estimate was CD4  = 0.12 IL-27 +332.8, *p* = 0.26. B. Linear regression of CD4+ against IL-27 in the HIV/HCV-co-infected group. In HIV/HCV-co-infected individuals, the CD4^+^ counts had a negative relationship with IL-27. The linear regresion estimate was CD4  = −0.13 IL-27 +493.4, *p*-value  = 0.29.

In the HIV-mono-infected group, CD4^+^ titers and IL-27 level had a positive correlation (*r* = 0.17, *p* = 0.13; Table 2). Linear regression of CD4^+^ and log HIV implies that for every 100 pg/ml increase of IL-27, the number of CD4^+^ monocytes would increase by 12 counts per micro liter (Figure 2A; *p* = 0.26). On the contrary, in the co-infected group, CD4^+^ and IL-27 titers had a tendency of negative correlation. CD4^+^ monocytes decreased by 13 counts for every 100 pg/ml increase of IL-27 (Figure 2B, *p* = 0.29). Because IL-27 actives naïve CD4^+^ T-cells [20], this difference implies that co-infection with HCV might have altered the expression profile of IL-27, disrupted the biological function of IL-27, or accelerated CD4^+^ T cell death.

### Low and high HIV and HCV viral load subjects

**Figure 3:**
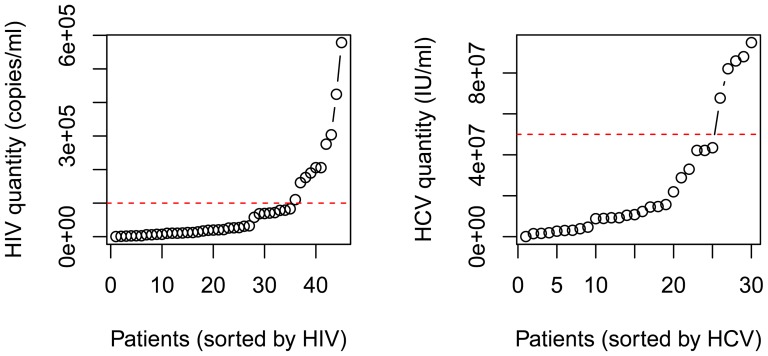
A. Dividing high and low HIV groups. The red dashed line marks HIV viral load of 10^5^ copies/ml, which was the separation place for the low and high HIV groups. B. Dividing high and low HIV groups. The red dashed line marks HCV viral load of 5×10^7^ IU/ml, which was the separation place for the low and high HCV groups.

All our subjects are in the early clinical stage of HIV-1 infection, with some predicted to be in acute infection due to the high viral loads determined. We aimed to analyze the relationship between IL-27 and HIV-1 viral load when excluding those acutely infected and where the relationship may be complicated [28]. To find the appropriate division point of low and high viral load, the HIV titers (copies/ml) of the mono-infected individuals were sorted and plotted in Figure 3A. It can be clearly seen that the trend significantly increased when the viral load was above 10^5^ virions/ml. The HIV level was divided into low HIV groups (35 subjects) and high HIV groups (eight subjects). Similar division was done on the co-infected group by cutoff at HCV level 5×10^7^ IU/ml (Figure 3B). The low HCV group had 25 individuals, and the high HCV group had five individuals.

### IL-27 was differentially associated with HIV titers in the HIV-mono-infected group and the HIV/HCV-co-infected group

**Figure 4:**
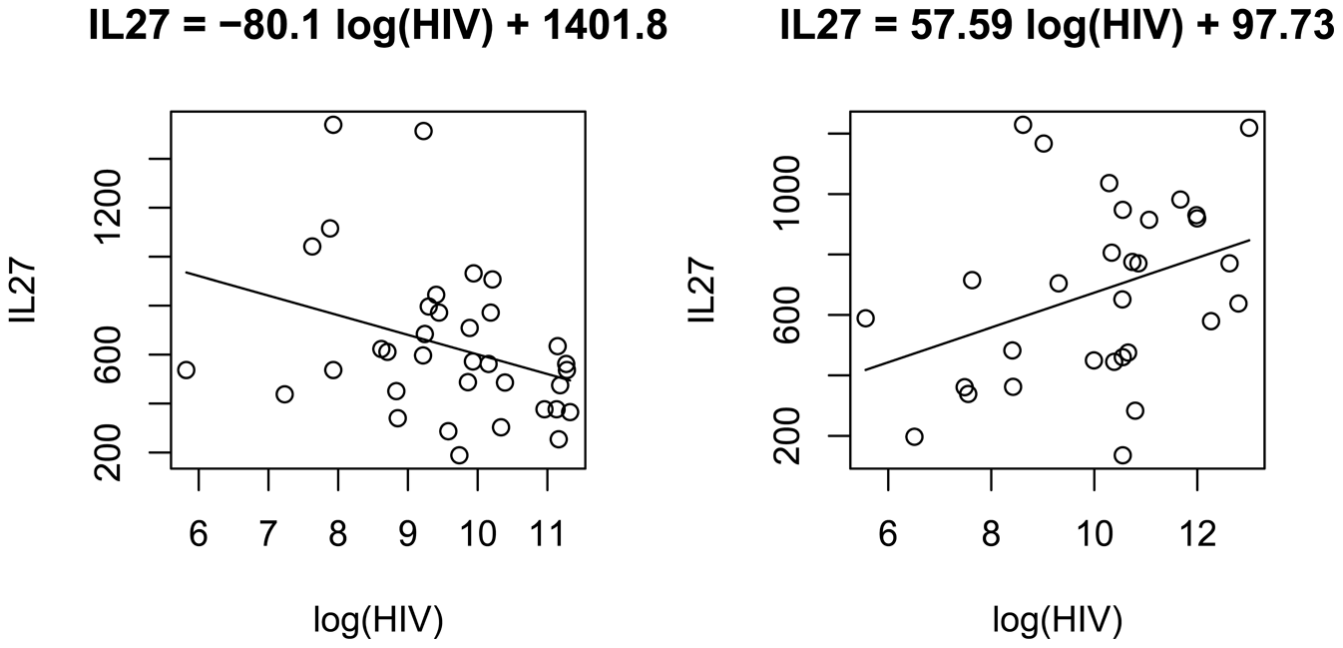
A. Linear regression of IL-27 on HIV viral load in low HIV-mono-infected subjects. In HIV-mono-infected individuals, the IL-27 had a negative relationship with natural log of HIV viral count. The linear regresion estimate was IL27  = −80.1 log(HIV) +1,401.8, and *p*-value  = 0.0485. B. Linear regression of IL-27 on HIV viral load in HIV/HCV-co-infected subjects. Contrary to the significant negative relation resulting in the HIV-mono-infected group, in the HIV/HCV-co-infected individuals the IL-27 had a positive relationship with the natural log of HIV viral count. The linear regresion estimate was IL27  = 57.59 log(HIV) +97.73, and *p* = 0.0495.

HIV was taken the natural log transformation for variance stablization, while IL-27 titers had a normal distribution and no adjustment is needed. In the mono-infected group, the IL-27 titers correlated to log HIV viral load negatively (*r* = −0.04, *p* = 0.3). In the low HIV-mono-infected group (35 subjects), IL-27 level negatively correlated with log HIV titers significantly (*r* = −0.34, *p* = 0.024). A scatter plot of IL-27 against log HIV was in Figure 4A (low HIV group); the regression *p*-value was 0.0485. On the contrary, in the co-infected group, the same pair of quantities had a significant positive correlation (*r* = 0.36, *p* = 0.025). The scatter plot was in Figure 4B, and regression *p*-value was 0.0495. In both the low HCV group and the low HIV group of the co-infected, the IL-27 and HIV titers had a positive correlation: the low HIV group had 23 subjects; the correlation co-efficient was 0.20 and the *p*-value was 0.18; the low HCV group had 25 subjects and the correlation co-efficient was 0.38 and the *p*-value was 0.029. In sum, the IL-27 level and the HIV viral load had a negative relationship in the HIV-mono-infected individuals and a positive relationship in the HIV/HCV-co-infected individuals.

## Discussion

In this study, we showed that plasma IL-27 titer was evidently elevated in HIV-positive individuals rather than in the health controls. This is consistent with previous reports describing the inhibition of HIV-1 replication by IL-27 and where the increased level of the cytokine is due to early stage immune response [19], [23], [25]. In the advanced stage of HIV infection, the virus may suppress the expression of IL-27 due to HIV-1 down-regulating immune responses [23]. There was no statistical difference in the average level of IL-27 in the HIV mono-infected and the HCV-co-infected subjects. In one previous study the suppression of IL-27 by HCV co-infection was observed, yet the study was conducted in different conditions with patients receiving HAART and the sample size was only nine [23]. The difference may result from different sample characteristics; this could be because our subjects were “healthy” (CD4^+^ counts ≥350 cells/µL) and were treatment-naïve. Our results support the report that HIV is the main target of immune responses under HIV/HCV co-infection circumstances [29].

This study is the first to report that IL-27 and CD4^+^ in treatment-naive individuals had a positive relationship in HIV-1 mono-infected individuals, whilst a negative relationship was described for HIV-1/HCV co-infected individuals. The positive relationship between IL-27 and CD4^+^ in mono-infected subjects was found by *in vitro* findings that IL-27 promoted the proliferation of naive CD4^+^ T-cells [11]. When a subject was infected with HIV, IL-27 was secreted to combat the invading HIV with its anti-inflammatory properties and to restore an intact immune system by boosting naïve CD4^+^ T cells [11], [15]–[17]. In this anti-HIV feedback arc set, IL-27 acts as the mediator; IL-27 elevates when HIV replicates, and it decreases when HIV replication is prohibited. However, in the HCV-co-infected group, the level of CD4^+^ negatively associated to IL-27, thereby meaning that under the HCV co-infection, the anti-HIV feedback arc set might be interrupted, which might result in the uncontrolled or less effective suppression of HIV replication [30]–[33]. Then, in an interrupted anti-HIV feedback arc set, high HIV viral load co-existed with high plasma IL-27 titer and low CD4^+^ T cell counts; this may explain the positive correlation between HIV viral loads and IL-27 titers in the HIV/HCV co-infection group and a native correlation trend between CD4^+^ counts and IL-27 titers. High IL-27 with high HIV viral loads among HIV/HCV-co-infected individuals might elevate liver-related morbidity and mortality; this is because IL-27 plays a key pathogenic role in T-cell-mediated hepatitis [8], [14], [34]–[36]. This study was based on samples from a previous voluntary-based HIV/AIDS surveillance study; the left plasma was not enough for us to evaluate liver enzyme alanine aminotransferase and aspartate aminotransferase levels. Although the subjects collected were asymptomatic HIV/HCV positive subjects without obvious liver damage, such as pancytopenia or portal hypertension, the accurate status of liver damage was not characterized. The lack of liver damage markers hindered our further analysis of HIV/HCV viral loads and IL-27 titers on liver damage progression. In this study, cross-sectional data on HIV/HCV viral loads, CD4^+^ T cell counts and IL-27 titers were taken, and, in future work, longitudinal data on these quantities with disease progression would provide greater insight into the cytokine response mechanism.

In sum, we confirmed that plasma IL-27 was significantly elevated in HIV-positive individuals. IL-27 was positively associated with CD4^+^, and negatively associated with HIV viral load in HIV-mono-infected individuals. However, under HCV co-infection, the regulation trends were completely altered. Our findings show that HCV co-infection altered the correlations between HIV viral loads, IL-27 titers and CD4^+^ T cell counts. In conclusion, our results suggest that IL-27 differs in treatment-naïve groups which have HIV mono-infections and HIV/HCV co-infections. This is critical information to be considered when caring and treating those with HIV mono-infection and HIV/HCV co-infection.
